# Influence of Abdominal Hollowing Maneuver on the Core Musculature Activation during the Prone Plank Exercise

**DOI:** 10.3390/ijerph17207410

**Published:** 2020-10-12

**Authors:** Miguel García-Jaén, Juan Manuel Cortell-Tormo, Sergio Hernández-Sánchez, Juan Tortosa-Martínez

**Affiliations:** 1 Area of Physical Education and Sports, Faculty of Education, University of Alicante, 03690 San Vicente del Raspeig, Spain; m.garciajaen@ua.es (M.G.-J.); juan.tortosa@ua.es (J.T.-M.); 2 Translational Research Center of Physiotherapy, Physiotherapy Area, Miguel Hernández University, 03550 Sant Joan d’Alacant, Spain; sehesa@umh.es

**Keywords:** core training, lumbar stabilization, bridging exercises, electromyography, physical therapy

## Abstract

This cross-sectional study of repeated measures investigated whether integrating the abdominal hollowing maneuver (AHM) into the prone plank performance is an effective strategy for increasing both the activation of the deep and superficial core musculature. Electromyographical (EMG) responses of rectus abdominis (RA), external oblique (EO), internal oblique (IO), and lumbar erector spinae (LES), and ratings of perceived exertion (RPE) of 20 participants (13 male, 7 female; mean ± standard deviation (SD) age: 24.25 ± 3.54 years; body mass: 66.42 ± 8.40 kg; height: 1.70 ± 9.51 m) were compared across two experimental conditions: the traditional prone plank (STANDARD); and a variation including the AHM (HOLLOWING). Regarding Total Intensity, HOLLOWING resulted in significantly greater EMG response than STANDARD (*p* < 0.001; Effect size (ES) = 3.01). Specifically, RA showed no significant differences between STANDARD and HOLLOWING (*p* = 0.056; ES = 0.285). However, for the remaining analyzed muscles, HOLLOWING significantly provided higher EMG activation compared to STANDARD (LES: *p* = 0.004; ES = 0.619; left EO: *p* < 0.001; ES = 1.031; right EO: *p* < 0.001; ES = 1.419; left IO: *p* < 0.001; ES = 2.021; right IO: *p* < 0.001; ES = 2.269). Regarding RPE, HOLLOWING reported values significantly greater than STANDARD (*p* < 0.001; ES = 2.94). In conclusion, integrating the AHM into the prone plank exercise enhances overall abdominal activity, particularly in both obliques. These findings provide updated guidelines for lumbar stabilization and core strengthening in health-related physical fitness programs.

## 1. Introduction

Physical fitness is important to public health as it aims to enhance the well-being, quality of life and health of the individuals throughout their entire lifespan [1,2,3]. The health-related components of physical fitness—muscular strength and endurance, cardiorespiratory fitness, flexibility, and body composition—are clearly involved in the daily-life activity or exercise of each person [1]. In recent years, research has pointed to core training as an essential component of health-related physical fitness programs [4,5,6,7,8,9]. The health benefits of core strengthening and stabilization are interrelated and include: improved stabilization, balance, and postural control (a stable and strong core assists lumbar spine and pelvis for maintaining the trunk stability, thereby decreasing perturbances in the neutral zone of spine) [10,11,12]; increased muscle power development and enhanced movement efficiency (a stronger core allows force and energy to be effectively transferred to the limbs through the kinetic chains) [6,13,14]; reduced risk of injury (a low degree on core strength can lead to a distal overload, causing injury on the limbs) [5,15,16]; and decreased risk of several musculoskeletal and back disorders (excessive load on lumbar spine, imbalances on lumbopelvic-hip complex, atrophy of paraspinal muscles…), which are consequences of impaired body postures and sedentary lifestyles [9,17,18].

Therefore, a well-trained core is a key component for rehabilitation of spinal and back disorders as well as for fitness or athletic enhancement and musculoskeletal injury prevention [5,9,17,19,20]. Optimal core function provides proximal stability for distal mobility by controlling the movement of the central region during motor tasks, and serves as a link for the effective transfer of force and energy between the trunk and limbs [6,21,22,23]. In contrast, insufficient fitness and impaired motor control of the core lead to increased instability of lumbar spinal segments and unfavorable distribution of loads into the lumbopelvic region, which are considered etiological factors of low back pain and other back disorders [17,18,24].

The core works synergistically as an integrated functional unit [7,22], which requires both passive stiffness (provided through the osseous and fascial structures) and active stiffness (achieved through muscular cocontraction), and is coordinated by the sensory-motor control system to maintain or regain the neutral zone of the lumbar spine during static and dynamic motor tasks [7,10,22,25]. Into this interdependent kinetic system, the lumbopelvic-hip complex musculature integrates both local and global synergistic muscle systems providing actively core stabilization [10,26,27]. The local stabilization system includes the deeper muscles—such as internal oblique (IO) and transversus abdominis (TrA)—that play a major role enhancing segmental control and stability of the lumbar spine [4,26]. The global stabilization system includes the larger superficial muscles—such as external oblique (EO), rectus abdominis (RA) and erector spinae—which provide torque across multiple segments for transferring the load directly between the thoracic cage-upper limbs and the pelvis-lower limbs [4,28]. It has been reported that increased vertebral stiffness by synergistic coactivation of both muscle systems is a central question improving the stabilization of the trunk [23,29,30].

Core stabilization and strengthening programs are aiming to promote both sensory-motor control and muscular strength and endurance of the lumbopelvic-hip region [7,10,23,31]. Consequently, appropriate trunk stabilization and strengthening exercises should aim to restore or enhance neuromuscular responsiveness needed to mechanical stabilization of the spine, emphasizing proper levels of muscular activation and stiffness through both muscle-system synergistic coactivation [7,8,10,17,23,25,31,32]. Within these exercises, both lumbar-spine stabilization maneuvers and abdominal bridging exercises have commonly been proposed as different strategies for retraining and strengthening the deep and superficial core muscles into the therapeutic practice or within fitness, conditioning and athletic settings [8,33,34,35,36,37].

On the one hand, lumbar stabilization maneuvers comprise different abdominal activation strategies, such as abdominal bracing (ABM) and abdominal hollowing or draw-in maneuvers (AHM), aiming to enhance the mechanical stability in the lumbar spine into the first stages in the therapy process when patients exhibit signs of lumbar segmental instability [35,37,38,39,40,41]. The AHM was designed to emphasize deep local muscle activity drawing-in the abdomen while the ABM contracts both local and global core muscles by pushing the abdomen out externally [35,36,37,38,40,41]. On the other hand, planks, or bridges exercises are frequently included as an important part of athletic, fitness, and rehabilitation programs. In fact, they have been pointed as essential exercises that programs in preventing lower limb injuries and back disorders should include [7,23,42,43]. Into bridging exercises, the traditional prone plank is a bodyweight exercise that causes the coactivation of core muscles increasing the intraabdominal pressure for providing functional stability to the spine [8,18,44].

Once deep muscular motor control is specifically retrained, this segmental stabilizing function should be integrated with the global trunk stabilizing function into subsequent more general core exercises to successfully reeducate and retrain overall trunk musculature in their essential stabilizing function prior to accepting greater loads in dynamic exercises [11,19,23,43]. Following these criteria, it would be interesting to integrate specific lumbar stabilization maneuvers into traditional bridging exercises. Interestingly, this dual-task performance could modulate the activity of both the deep and superficial muscle systems, offering novel insights for reeducating, retraining, or strengthening the core. Many studies have been performed to analyze the different effects of the isolated AHM in various positions—supine, prone, standing, side-lying, and quadruped—showing an emphasized activation of the local muscle system [34,35,37,38,39,40]. However, to the best of our knowledge, little evidence of including these hollowing effects along with the bracing effects of the traditional prone plank exercise is available. As a result, it would be necessary to define more clearly the possible influence of this lumbar stabilization maneuver modulating the core activation during this essential bridging exercise. Therefore, the purpose of this study was to investigate whether integrating the AHM is an effective strategy to enhance the activation of the deep and superficial core muscles when performing the traditional prone plank exercise. It was hypothesized that including the AHM during the prone plank performance would provide greater muscle activation responses, specifically in the local core muscles evaluated, while the global musculature analyzed would maintain similar activity.

## 2. Materials and Methods

### 2.1. Experimental Design

**Figure 1:**
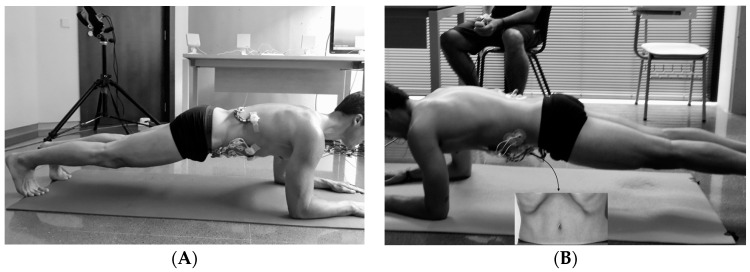
Graphical representation of the two prone plank variations. (**A**) STANDARD experimental condition; (**B**) HOLLOWING experimental condition.

A cross-sectional study of repeated measures was designed aiming to analyze the effects of performing the AHM on the muscle activation of four core muscles—RA, EO, IO and the lumbar portion of erector spinae (LES)—while performing the prone plank exercise. Electromyographical (EMG) responses and ratings of perceived exertion (RPE) of two experimental conditions were randomly collected and compared: the traditional or standard prone plank exercise (STANDARD); and a prone plank variation based on the STANDARD, with the simultaneous incorporation of the AHM (HOLLOWING). Figure 1 presents the different experimental conditions evaluated.

### 2.2. Participants

A convenience sample of 20 healthy and physically active college students (13 male, 7 female), were selected to participate in this investigation (mean ± standard deviation (SD) age: 24.25 ± 3.54 years; body mass: 66.42 ± 8.40 kg; height: 1.70 ± 9.51 m; abdominal skinfold thickness: 12.23 ± 2.45 mm; supra-iliac skinfold thickness: 10.36 ± 1.82 mm). Inclusion criteria comprised having previous experience in resistance and core training (4.45 ± 1.73 years); performing at least three physical-exercise sessions per week at moderate to vigorous intensity (4.70 ± 1.66 sessions); no history of musculoskeletal or central nervous system (CNS) disorders; no abdominal, hip or low back surgeries; and no history of acute or chronic pain or injuries located around knees, hips, spine, elbows, shoulders, or neck into last year. Furthermore, previous experience performing the prone plank and the AHM was requested. Additionally, participants with a skinfold thickness in the electrode placement area greater than 20 mm were also excluded from study, for decreasing the eventual EMG artifact due to subcutaneous adipose tissue [35,45]. All selected participants provided written informed consent as required for protection of human participants.

### 2.3. Procedures

#### 2.3.1. Study Protocol

Two different sessions, separated between 48–72 h, were carried out for each participant: familiarization and experimental sessions. All measurements were made by the same researchers at the same university laboratory. During the familiarization, participants were informed about several mandatory restrictions for the experimental session: no food and drinks consumption, or medication or any CNS-stimulants used within 2–4 h before data collection; no training or performing any moderate or vigorous physical activity 24–48 h before measurements; and sleep at least 7–8 h the night before the experimental session. All protocols and experimental procedures of this investigation comply with the ethical principles of research with human beings, as described in the 64th General Assembly of the Declaration of Helsinki in 2013. This article adheres to the STROBE (*Strengthening the Reporting of Observational studies in Epidemiology*) guidelines [46]. The research protocol was approved by the University Ethics Committee (UA-2018-11-16).

#### 2.3.2. Familiarization Session

72–48 h before their experimental session, participants performed a preliminary familiarization session at the same time and the same laboratory where they would later perform the data collection session, aiming to set up the right performance of all experimental conditions and become acquainted with the measurement instruments and procedures. Anthropometrical measurements were collected at the beginning of this session by an expert anthropometrist (III-Level ISAK—*International Society for the Advancement of Kinanthropometry*—). Body mass and height were measured using a balance beam scale (Avery Ltd. Model 3306 ABV) and a stadiometer (Holtain Ltd.), respectively. Skinfold thickness assessment was performed using an approved body fat caliper (Holtain Ltd.) following the standardized ISAK guidelines [47].

After a 10 min warm-up, consisting of mild cardiovascular exercise and joint mobilization, participants were individually trained, supervised and corrected by a Certified Strength and Conditioning Specialist (NSCA—CSCS) about the proper AHM and prone plank performance. Then, they were tutored to practice both experimental conditions, improving their perception of adequate task control throughout the use of verbal and manual feedback. After the familiarization, only those who were able to correctly perform these tasks continued into the study. Ending this session, participants were informed about the aforementioned restrictions for the subsequent data collection session. The session length ranged 40 to 60 min depending on participants.

#### 2.3.3. Abdominal Hollowing Maneuver and Prone Plank Protocols

Table 1 summarizes the AHM and prone plank protocols, already stablished in previous studies [35,48,49,50,51,52]. Both exercises were closely monitored ensuring its correct performance into a learning-process to improve its control and body awareness. The AHM was first performed in stand-up and prone-lying positions, and secondly integrated in the prone plank. The maneuver was closely controlled to ensure that no pelvic tilting backward occurred while performing these tasks. Additionally, a pressure biofeedback unit—PBU— (Stabilizer, Chattanooga Group Inc., Hixson, USA) was also placed under the abdominal wall during the prone-lying position, providing a complementary sensitive and visual feedback for helping participants to control the AHM properly. Measurements from PBU have been used in previous research enhancing participants’ insight into their deep abdominal muscle recruitment and thereby increasing their correct maneuver control and learning [53,54]. All participants were required to practice both AHM and the different plank protocols under supervision, for as many times as needed until they felt confident of understanding and controlling the performance these tasks.

#### 2.3.4. Experimental Session

Surface EMG on the testing muscles RA, left external oblique (LEO), right external oblique (REO), left internal oblique (LIO), right internal oblique (RIO) and LES were collected simultaneously while randomly performing the STANDARD or the HOLLOWING experimental conditions. RPE, using the OMNI-Resistance Exercise Scale (OMNI-RES) [55], was also collected immediately after each experimental condition.

Participants performed three sets of 10 s isometric contractions of each condition, from a neutral performance position controlled by two researchers. They were instructed to maintain this position during measurements, since varying joint positions could influence the EMG response of the muscles analyzed [51,52]. The order of each condition was randomized using a balanced Latin Square approach, minimizing any potential confounding effects of exercise sequence on results [51,52]. Participants rested for 5 min between exercises minimizing the possibility of residual fatigue.

#### 2.3.5. Maximal Voluntary Isometric Contraction and EMG Data Collection

**Figure 2:**
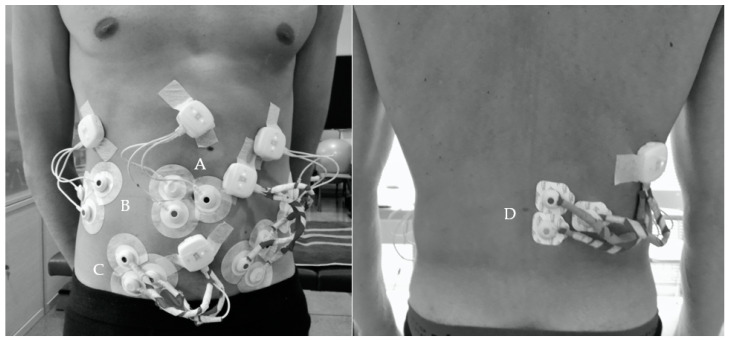
Bipolar surface electromyographic electrode and wireless sensor placement. (**A**) Rectus Abdominis: parallel to the rectus abdominis muscle fibers and approximately located 3 cm lateral and across from the umbilicus over the muscle belly. (**B**) External Oblique: parallel to the external oblique muscle fibers and approximately located 15 cm lateral and across from the umbilicus. (**C**) Internal Oblique: parallel to the internal oblique muscle fibers and halfway between the anterior superior iliac spine of the pelvis and the midline, just superior to the inguinal ligament. (**D**) Erector Spinae: approximately 3 cm from the L3 spinous process, over the muscle belly and parallel to the erector spinae muscle fibers.

The EMG signal of the RA, LEO, REO, LIO, RIO and LES during the different conditions was recorded for 10 s by telemetry using the Mega Wireless Bio Amplifier (WBA^®^) system (Mega Electronics, Kuopio, Finland). The EMG signals were collected using pre-gelled disposable bipolar Ag-AgCl surface electrodes placed parallel to the muscle fibers with a center-to-center spacing of 20 mm [56]. Before electrode placement, the overlying skin area of testing muscles was shaved and cleaned with alcohol cotton wipes. Electrodes were placed on the right side, for the RA and LES, and bilaterally for the EO and IO, in accordance to accepted international guidelines of *Surface EMG for Non-Invasive Assessment of Muscles* (SENIAM) project [56]—and if not available, according to previous research [11,57]. Figure 2 shows in detail the electrode and wireless sensor placement procedure. Skin marking through manual palpation was performed using a dermal skin marker to accurately place the electrodes [58]. Once the electrodes were placed, participants were asked to perform different movements to test the signal quality. EMG signal was converted from analogue to digital using an A/D converter (National Instruments, New South Wales, Australia). Data were registered with MegaWin software package (MegaWin^®^ 3.0; Mega Electronics LTD) with a sampling frequency of 1000 Hz and were bandpass filtered (12–450 Hz) using a fourth-order Butterworth filter. Then, 10 s average root-mean-square (RMS) value for each muscle was calculated using the LabVIEW software (National Instruments, Austin, TX, USA). Time window length of moving RMS method was set at 500 milliseconds for calculation of EMG average RMS values. Apart from the EMG signal of each individual muscle, the overall EMG activity (total intensity, TI) elicited during each exercise was also analyzed. TI was defined as the sum of the normalized EMG value of all six muscles, as previously measured in a previous investigation [51].

The collected EMG data were normalized as a percentage of a maximal voluntary isometric contraction (MVC). For this purpose, two MVC were performed against manual resistance for each muscle before the plank exercises and after a 5 min standardized warm-up. The detailed MVC protocol has been described in previous research [59]. For the abdominal muscles, participants produced maximal isometric efforts in trunk flexion for the RA (Intraclass Correlation Coefficient (ICC) = 0.999), and also in lateral bend and twist for both EO (ICC = 0.998 for the LEO and 0.999 for the REO) and both IO (ICC = 0.999 for the LIO and 0.999 for the RIO), respectively. For the LES, maximal isometric trunk extensions were performed in the Biering–Sorensen position (ICC = 0.999). Each MVC was maintained for 5 s and a 5 min rest was allowed between sets, minimizing neuromuscular fatigue. The average RMS value of the two MVC sets was considered the reference value for EMG normalization.

#### 2.3.6. Perceived Exertion Data Collection

A printed copy of the OMNI-RES scale providing visual feedback was also used as a complementary measure of effort for each prone plank performed. Each RPE was collected as a step-by-step process after each experimental condition performed, ensuring that the perceived exertion was only referred to this condition. All participants already knew the scale and additionally they were skilled with the RPE reporting procedures at the familiarization session.

### 2.4. Statistical Analyses

Statistical analyses were performed with SPSS 24.0 (SPSS Inc. Chicago, IL, USA). After checking the normality of the data (Kolmogorov–Smirnov), an analysis of variance (ANOVA) was used to compare the differences in the activation of the different muscles measured during each condition, and a paired samples t-test was used for the differences between the two plank conditions (STANDARD vs. HOLLOWING) in each muscle group. Post hoc power analysis was performed for t-test family tests for differences between two dependent means using the G*Power 3.1.9.2 (Heinrich–Heine Universität, Germany), where significant differences were found between interaction effects [60]. The reliability of the MVC tests and exercises was determined by a 2-way random effect model ICC with 95% confidence intervals (CI). Statistical significance was set at *p* ≤ 0.05. Effect size (ES) was estimated with Hedges *g* [61], using the following scale: <0.2 = trivial; 0.2–0.5 = small; 0.5–0.8 = medium; 0.8–1.3 large; and >1.3 very large. All variables are reported as mean ± SD.

## 3. Results

### 3.1. Differences Across Core Musculature

Mean and SD values of the normalized EMG muscle activity, ICC and 95% CI, for the RA, LES, LEO, REO, LIO, and RIO, across the two prone plank variations analyzed are presented in Table 2. For the STANDARD condition, no significant differences on EMG activation levels were found across all abdominal muscles evaluated (RA-LEO, *p* = 0.541; RA-REO, *p* = 0.264; RA-LIO, *p* = 0.381; RA-RIO, *p* = 0.737; LEO-REO, *p* = 0.656; LEO-LIO, *p* = 0.828; LEO-RIO, *p* = 0.768; REO-LIO, *p* = 0.809; REO-RIO, *p* = 0.433; LIO-RIO, *p* = 0.588). However, the EMG activation level of the back region, measured in the LES, was significantly lower in this STANDARD condition compared to the rest of the abdominal muscles (*p* < 0.001).

For the HOLLOWING condition, statistical differences on EMG activation were observed across the abdominal musculature evaluated. Specifically, the LIO and RIO provided higher values compared to the rest of the abdominal muscles (LIO-RA, *p* < 0.001; LIO-LEO, *p* < 0.001; LIO-REO, *p* < 0.001; and RIO-RA, *p* < 0.001; RIO-LEO, *p* = 0.001; RIO-REO, *p* < 0.001). The EMG response in RA and EO muscles showed no significant differences into this condition (RA-LEO, *p* = 0.082; RA-REO, *p* = 0.135). For the EO and IO, no significant differences were observed into both sides of each oblique muscle (LEO-REO, *p* = 0.741; LIO-RIO, *p* = 0.883). The LES activation was significantly lower when compared to each abdominal muscle analyzed (LES-RA, *p* = 0.003; LES-LEO, *p* < 0.001; LES-REO, *p* < 0.001; LES-LIO, *p* < 0.001; LES-RIO, *p* < 0.001).

### 3.2. Differences Across Experimental Conditions

**Figure 3:**
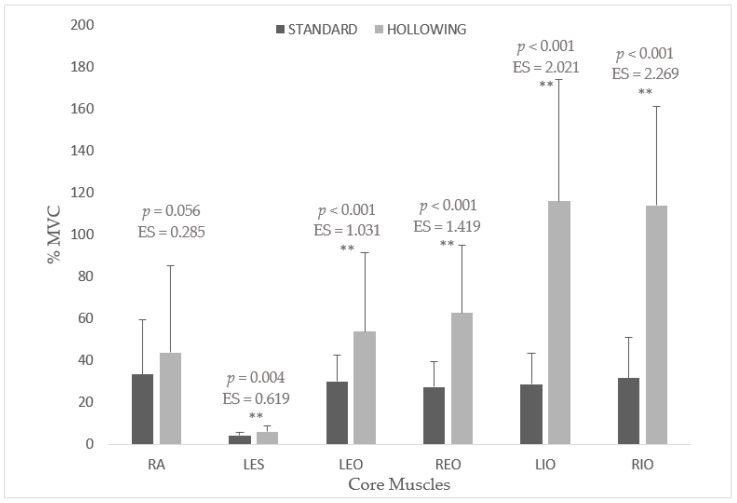
Comparison of normalized electromyographic activity (%MVC) of the core muscles across experimental conditions. Abbreviations: RA, rectus abdominis; LES, lumbar portion of erector spinae; LEO, left external oblique; REO, right external oblique; LIO, left internal oblique; RIO, right external oblique; STANDARD, prone plank standard condition; HOLLOWING, prone plank hollowing condition; ES, Effect Size. ** Significant differences on EMG muscular response between both prone plank conditions.

Differences on normalized EMG activation for all core musculature analyzed during both experimental conditions are exposed in Figure 3. For the RA, no significant differences between STANDARD and HOLLOWING were observed. Regarding the LES, HOLLOWING resulted in significantly greater EMG response than STANDARD. For the EO musculature, the HOLLOWING condition provided a significantly higher EMG activation compared to the STANDARD condition. A significantly greater EMG activity was also found in the IO musculature for the HOLLOWING condition. Specifically, in comparison to STANDARD, HOLLOWING elicited significant higher muscle activity in LIO and RIO. The values obtained in the post hoc power analysis were: 0.825 to LES; 0.989 to LEO; 0.999 to REO; 0.999 to LIO; 1.000 to RIO.

### 3.3. Total Intensity and Rated Perceived Exertion

Mean and SD values of RPE and TI, expressed in %MVC, across experimental conditions are shown in Table 3. In terms of TI, HOLLOWING showed significantly greater EMG activation in overall musculature compared to STANDARD (*p* < 0.001; ES = 3.01). Regarding RPE, the HOLLOWING values reported by participants were significantly higher than the reported STANDARD values (*p* < 0.001; ES = 2.94). The values obtained in the post hoc power analysis were: 1.000 to RPE; 1.000 to TI.

## 4. Discussion

This study aimed to investigate whether integrating the AHM when performing the prone plank exercise is a more effective strategy enhancing overall trunk muscle activity compared to traditional prone plank performance. Results partially supported our hypotheses and showed that the AHM-prone plank provided significantly higher RPE and EMG responses in participants, both in terms of TI and specifically within IO local muscles, while RA and LES global muscles maintained similar activity levels. However, contrary to our initial hypothesis, the AHM-prone plank also resulted in significant increases in activation on EO global muscles, although not to the same extent as the changes observed in IO muscles. These findings highlight the greater recruitment both local and global abdominal musculature for contributing to the trunk stabilizing and weight-bearing demands by including the AHM during the traditional prone plank performance. This enhanced activation is particularly significant not only in the deep oblique musculature, but also in the more superficial obliques, suggesting a greater trunk stabilization effect by increasing the active muscular stiffness of the core [4,28,41].

### 4.1. Comparison of EMG Activity Across Core Muscles

For classifying muscle activation as low-to-high levels the criteria used in previous studies were applied [62,63]. The STANDARD condition elicited similar amplitudes of moderate activity across all abdominal muscles. However, there was no variation in the amplitude of EMG activity on the back region, measured in the LES, that remained low for both experimental conditions. These results are consistent with previous research investigating this exercise, thereby indicating a similar performance of the traditional prone plank [34,39,52,63].

However, the abdominal EMG activity varied importantly when performing the HOLLOWING condition. First, both RA and EO reached high levels of muscle activation, without differences in the muscle response between these global muscles. This effect of enhanced muscle recruitment may be partially elicited by performing the AHM integrated in the plank on a prone position [64] and differs between both superficial muscles. In contrast to the RA, which only increased its activation around 10% MVC from STANDARD by incorporating the AHM, both EO showed higher responses by increasing two-fold its activity—from around 25% MVC to more than 50% and 60% MVC in LEO and REO, respectively—. As it can be noted, the EO muscles were most active with this dual-task integration. This point remains controversial in related literature investigating the EO activity during the AHM. Although some studies suggested that the AHM mainly elicits the recruitment of the deeper IO/TrA muscles with minimal activity of more superficial EO/RA muscles in isolated drawing-in maneuvers performed in starting supine and prone positions [35,65], other studies stated that the EO activity is unfeasible to suppress when the AHM is performed in prone, maintaining low to moderate activation levels [41,48,64]. Furthermore, to the best of our knowledge, only a pilot-study has examined a four-kneeling bridging position with hollowing instructions, reaching around 40% MVC of the EO activation [66]. The discrepancies about our EO results could be due to the differences between exercises and protocols among studies. Additionally, their sample included participants reporting mild-to-moderate low back pain, while all our participants were asymptomatic. The presence of pain might have altered the ability of participants to drawing-in their abdomen during tasks [67].

Secondly, the greatest EMG response by merging the AHM into the prone plank was observed for the deep IO muscles, compared to the back region and the rest of the superficial abdominal wall. This response was very high and almost identical between both IO sides, reaching activation values over 110% MVC. This may be due to the effort supplying the stabilizing and weight-bearing demands for this dual-task exercise, and it is consistent with previous reports of greater IO activity performing the AHM during different tasks [35,37,48,64,66]. Our results are slightly higher but coherent with these findings supporting the idea that the IO, probably along with the TrA, plays a large role in creating the AHM. Although this regard was not directly measured in this study due to the use of surface EMG, it has been considered that the lower fibers of the IO run parallel to the TrA, suggesting that they function in a similar way [68]. In any case, according to the aforementioned research, our results suggest that performing the AHM along with the prone plank induces a higher activation effect in the deep IO musculature.

### 4.2. Comparison of EMG Activity Across Experimental Conditions

The magnitude of the core EMG responses differed between both testing conditions. For the STANDARD condition, the obtained values were similar to those reported in previous studies [34,39,52,63]. However, when participants were asked to perform simultaneously the AHM, they experienced a significant intensification in overall core coactivation. This EMG increase, expressed in %MVC, for each muscle was: 10.64% in the RA, (ES = 0.285); 1.60% in the LES, (ES = 0.619); 29.80% in the LEO, (ES = 1.031); 35.30% in the REO, (ES = 1.419); 87.24% in the LIO, (ES = 2.021); 82.58% in the RIO, (ES = 2.269). In terms of TI, the merged muscle activity significantly raised by 40.53% (ES = 2.896), with the oblique musculature representing the largest proportion of this shift on core muscle recruitment. Accordingly, the RPE informed by participants also increased two-fold, showing a variation of 3.31 points in the OMNI-RES scale.

All these findings corroborate our initial hypotheses indicating that the AHM integrated in the prone plank effectively influenced and enhanced the neural activity of overall core musculature as well as modulated the perceived exertion of participants compared to the traditional prone plank. In particular, this dual task strongly encouraged the recruitment of the deep obliques and properly fostered the contraction of the superficial obliques, while promoting small or no additional effect on the global RA and LES muscles—despite the statistical significance found in the LES, this %MVC variation was minimal. Therefore, it seems that HOLLOWING, instead of producing any dual-task interference phenomenon [36], would require complementary recruitment of both core muscle systems, resulting in enhanced muscular stiffness and force production. Since it was emphasized that active force production is essential to provide stiffness, to stabilize and unload the spinal column [22,41,62], this combination could be recommended as a proper stabilization exercise oriented to rehabilitation and strengthening strategies.

### 4.3. Neuromuscular Recruitment Foundations and Hypotheses

The IO, along with the TrA, are considered to be key abdominal muscles of the local stabilizing system because their role providing active stiffness for stabilizing the trunk [6,21,29,69]. As suggested, there is often an IO/TrA coactivation for lumbar segmental stabilization, supported by several facts: first, the IO has both superficial and deeper layers sharing common attachments with the TrA through the thoracolumbar fascia [64,70,71]; Secondly, the architecture of the TrA suggests a limited capability providing spinal stability on its own, thereby needing the IO support [38,68]. Thus, although the TrA may be activated independently at very low levels of challenge (<5% MVC), at higher levels of activation acts as synergistic of the IO for improving the efficacy of the task [24,29,35,38]. Consequently, although the TrA activation could not be directly measured with surface EMG in our study, this IO/TrA synergistic stabilizing role during exercises could be assumed.

Within oblique musculature, the EO would be less affected by the AHM than the IO because is a more global torque-producing muscle, less involved in segmental spinal stability [68]. However, interestingly, the medial fibers of the EO are anatomically related to the thoracolumbar fascia and thus biomechanically would have some stabilizing role as well [27,72]. As a result, both obliques and the TrA inevitably would work together to flatten the abdomen during the AHM [35]. Additionally, research showed that the prone position affects the abdominal muscle recruitment when performing the AHM increasing the activity of the EO possibly by the greater gravitational demands or reflex-mediated activity of these superficial muscles in response to stretch during the AHM [48,64]. Also, moderate activation of the EO has already been observed performing the standard prone plank [34,39,52,63]. All these facts could help to explain the greater recruitment of both EO in our study, despite not activating to the same extent reached by the IO muscles. Finally, the RA shares minimal attachments with deeper abdominal muscles and has independent activation to the IO/TrA [35], explaining the minimal variation of 10% MVC across conditions. This is important because other essential criterion determining the effectiveness of the AHM is the minimal activation of the RA [35].

Considering all these foundations, the present findings reasonably suggest that performing the AHM-prone plank seems to produce a binding on the abdominal muscle recruitment contracting synergistically both local and global systems to jointly provide enhanced active stiffness. Since both abdominal systems play together an important role in achieving the spinal stiffness and the stabilization of the trunk [29,38,41,69], this dual-task exercise, rather than isolated tasks to hollow or bridging, could be recommended as a suitable strategy to enhance the muscular stiffness needed to mechanical stabilization of the spine.

### 4.4. Methodological Limitations and Future Research

First, this study used surface EMG electrodes for measuring activity from the deep abdominal muscles. Therefore, because of the cross-talk phenomenon [64], the EMG signal might have been obtained from both IO/TrA muscles. However, this should not affect study results, because both muscles have been proven to function synergistically during the AHM and the prone plank [24,29,35,38,39,70]. Nevertheless, more research should be addressed to directly evaluate the TrA activation in the AHM-prone plank exercise.

Secondly, this study was performed on young and healthy people with no low back disorders or pain. Although similar results could be expected into the symptomatic population, maybe the presence of pain or back injuries could result in different muscle activation patterns [67]. Consequently, our findings are limited to healthy participants and cannot directly extrapolate for clinical populations. Therefore, since the AHM can elicit spinal stiffness [41] and its integration in the prone plank could make the exercise more adequate for patients with back pain and signs of spine instability, further studies exploring the effects of the AHM-prone plank in symptomatic individuals with non-specific chronic low back pain need to be conducted.

Thirdly, future research should explore the effects on muscle activation by integrating the AHM during other dynamic core and resistance exercises. Additionally, future studies should investigate the long-term effects of a core training program using the AHM-prone plank exercise on the trunk muscle activity, including an adequate follow-up and other outcome measures related to the quality of life, well-being, and health of the participants.

### 4.5. Practical Applications

This study provides empirical evidence that including the AHM into the standard prone plank exercise can be an effective strategy for increasing overall abdominal activity. In particular, this dual task encourages the activation of the IO and causes an important shift of activation in the EO, with small effect on the moderate RA activation caused by prone plank. Thus, this AHM-plank variation elicits optimal muscle-recruitment levels across the local and global stabilizing muscle systems. Our findings offer insight into the additive effect of the AHM in selectively stimulating deep musculature although the prone plank would elicit a moderate recruitment in both muscle systems, due to the need to maintain the spine against gravitational forces. These findings provide new and updated guidelines to strength and conditioning specialists and physical therapists for core stabilization and strengthening training in athletic and rehabilitation settings.

Previous research has shown how the global abdominal musculature have been extensively trained inducing imbalances, especially in patients with low back disorders who tend to increase global-system muscle activity [39,44]. In fact, most core exercises cannot highly activate the local stabilizing muscles, which only have been selectively recruited by isolated lumbar stabilization tasks aiming to improve the neuromuscular control and function of deep core muscles [34,36,39]. Considering these evidence-based remarks, it should be recommended core stabilization exercises joining lumbar stabilization maneuvers through bridging tasks, as therapeutic possibilities for lumbopelvic stabilization during the different stages of core rehabilitation and core strengthening physical fitness programs. This new approach could be potentially useful for core reeducation in these health-related physical fitness programs and adds new insights into the controversy about the optimal strategy to retrain core muscles [73].

## 5. Conclusions

Integrating the AHM into the traditional prone plank exercise is an effective strategy for increasing overall abdominal activity, particularly in the internal and external oblique musculature, eliciting optimal activity levels across the local and global stabilizing systems. These findings provide updated guidelines to strength and conditioning specialists and physical therapists for core stabilization training in athletic and rehabilitation physical fitness programs aiming to enhance the well-being, quality of life, and health of their clients and patients.

## Figures and Tables

**Table 1:** Description of the AHM and the prone plank protocols.

Exercise	Task Protocol
Traditional Prone Plank Exercise	Lie face-down with fists on the floor, feet shoulder width apart, and spine, scapulae, pelvis, and head in neutral positions. The elbows spacing shoulder width apart directly below the glenohumeral joint. Lift the body up on the forearms and toes
Abdominal Hollowing Maneuver	Draw the navel in and up while not allowing any movement at the spine, rib, or pelvis and then holding the abdominal contraction for 10 s while breathing normally

**Table 2:** Muscle activity (%MVC) in different prone plank conditions.

	Prone Plank Conditions						
	STANDARD				HOLLOWING		
Muscles	Mean ± SD	95% CI	ICC		Mean ± SD	95% CI	ICC
Rectus Abdominis	33.20 ± 26.23	20.93– 45.47	0.986		43.84 ± 41.25	24.54–63.15	0.977
Lumbar Erector Spinae	4.28 ± 1.49 *	3.58–4.98	0.968		5.87 ± 3.00 *	4.47–7.28	0.963
Left External Oblique	29.84 ± 12.44	23.21–36.47	0.975		53.67 ± 37.57	36.08–71.25	0.995
Right External Oblique	27.39 ± 12.04	21.75–33.02	0.946		62.68 ± 32.46	47.49–77.87	0.991
Left Internal Oblique	28.64 ± 14.86	21.69–35.60	0.982		115.89 ± 58.29 †	88.61–143.17	0.985
Right Internal Oblique	31.46 ± 19.32	22.42–40.50	0.992		114.04 ± 47.26 †	91.92–136.16	0.970

* Significantly lower compared to the rest of the abdominal muscles, both in STANDARD condition and in HOLLOWING condition. † Significantly higher compared to the rest of the abdominal muscles. Abbreviations: MVC, maximal voluntary isometric contraction; SD, Standard Deviation; CI, Confidence Interval; ICC, intraclass correlation coefficient; STANDARD, traditional prone plank; HOLLOWING, traditional prone plank with abdominal hollowing maneuver.

**Table 3:** Comparison of Total Intensity (%MVC) and RPE between prone plank conditions.

	Total Intensity			RPE			
Prone Plank Conditions	Mean ± SD	95% CI		Mean ± SD	95% CI		ICC
		Low	High		Low	High	
STANDARD	23.77 ± 10.24	18.98	28.56	3.22 ± 1.28	2.62	3.82	0.966
HOLLOWING	64.30 ± 16.89 *	56.39	72.21	6.53 ± 1.37 †	5.90	7.18	0.972

* Significantly higher compared to STANDARD. † Significantly higher compared to STANDARD. Abbreviations: RPE, rating of perceived exertion; SD, Standard Deviation; CI, Confidence Interval; ICC, intraclass correlation coefficient; STANDARD, traditional prone plank; HOLLOWING, traditional prone plank with abdominal hollowing maneuver.
